# Evaluation of the Effects of *Euglena gracilis* on Enhancing Immune Responses in RAW264.7 Cells and a Cyclophosphamide-Induced Mouse Model

**DOI:** 10.4014/jmb.2212.12041

**Published:** 2023-01-20

**Authors:** Kyeong Ah Jo, Kyeong Jin Kim, Soo-yeon Park, Jin-Young Jeon, Ji Eun Hwang, Ji Yeon Kim

**Affiliations:** 1 Department of Food Science and Technology, Seoul National University of Science and Technology, Seoul 01811, Republic of Korea; 2 Department of Nano Bio Engineering, Seoul National University of Science and Technology, Seoul 01811, Republic of Korea; 3 BIO R&D Center, Daesang Corp., Seoul 07789, Republic of Korea

**Keywords:** Immune enhancement, *Euglena gracilis*, β-glucan, cyclophosphamide, splenocytes, macrophage

## Abstract

In this study we evaluated the immune-enhancing effects of β-glucan, the main component of *Euglena gracilis* (*Euglena*), and *Euglena* on inflammatory factor expression in RAW264.7 macrophages and ICR mice with cyclophosphamide-induced immunosuppression. Macrophages were treated with β-glucan or *Euglena* for 48 h. The β-glucan and *Euglena* groups exhibited higher levels of inducible nitric oxide synthase, nitric oxide, and tumor necrosis factor (TNF)-α than the control (vehicle alone) group. Animals were fed saline and β-glucan (400 mg/kg body weight (B.W.)) or *Euglena* (400 or 800 mg/kg B.W.) for 19 days, and on days 17–19, cyclophosphamide (CCP, 80 mg/kg B.W.) was administered to induce immunosuppression in the ICR mouse model. CCP reduced the body weight, spleen index, and cytokine expression of the mice. To measure cytokine and receptor expression, splenocytes were treated with concanavalin A (ConA) or lipopolysaccharide (LPS) as a mitogen for 24 h. In vivo, ConA stimulation significantly upregulated the expression of interferon (IFN)-γ, interleukin (IL)-10, IL-12 receptor β1, IL-1β, and IL-2 in splenocytes from the β-glucan- or *Euglena*-treated groups compared with those in the splenocytes from the CCP-treated group; LPS stimulation increased the levels of the cytokines TNF-α, IL-1β, and IL-6 in splenocytes from the β-glucan- or *Euglena*-treated groups compared with those from the CCP-treated group, but most of these differences were not significant. These results demonstrate the effect of *Euglena* in ameliorating macrophages and immunosuppression in CCP-treated mice. Thus, *Euglena* has the potential to enhance macrophage- and splenocyte-mediated immune-stimulating responses.

## Introduction

An immune response of the human immune system is necessary to protect our bodies from pathogens [1]. The main organs or tissues related to the immune system are bone marrow, the spleen, and lymph nodes. In particular, the spleen is the center of the mononuclear phagocyte activity system and contains lymphocytes, or splenocytes, that cooperate to ultimately promote antibody production [2, 3]. Macrophages and T cells are types of splenocytes. Macrophages secrete inflammatory cytokines, such as nitric oxide (NO), inducible nitric oxide synthase (iNOS), interleukin (IL)-1β, IL-6, and tumor necrosis factor-α (TNF-α) to promote the removal of external pathogens by the innate immune system via phagocytosis [4, 5]. In addition, macrophages play an important role in linking innate and adaptive immunity by presenting and delivering antigens to T cells to initiate adaptive immune responses [6]. Helper T (Th) cells are activated by IL-2. Th cells activate cytotoxic T cells and memory B cells, leading to an immune response cascade. The specific adaptive immune response promoted by Th cells depends on their subtype, and these subtypes are distinguished by the types of cytokines, such as interferon (IFN)-γ, IL-2, and IL-10, produced by the cells [7]. Immune deficiency occurs when the components of the immune system are destroyed [8] and can cause diseases such as chronic cough, lung infections, and diarrhea. Furthermore, in light of the COVID-19 pandemic, research on the development of immune-related functional ingredients is actively ongoing [9].

*Euglena gracilis* (*Euglena*) is a microalga that contains a variety of nutrients and minerals and has thus been used as a food supplement that promotes health [10]. Paramylon, a substance unique to euglenoids, is a β-1,3-glucan. *Euglena* accumulates paramylon for energy and carbon storage [11]. Under heterotrophic growth conditions, *Euglena* contains water-insoluble paramylon at levels that exceed 50% of its dry weight [12]. Similar to other β-1,3-glucans, paramylon exerts various bioactive effects on health, such as antitumor, anti-HIV, and inhibitory effects on fat accumulation [11, 13, 14]. In addition, previous studies on the effects of paramylon on immunity have shown that paramylon increases immune responses, such as phagocytosis, antibody production, natural killer cell cytotoxicity, and cytokine production [15].

A previous study on the mechanism through which *Euglena* enhances immune responses confirmed that it affects dectin-1 expression, cytokine gene expression, and natural killer (NK) cell activity in an immunosuppression model [16]. In this study, we further confirmed the effects of β-glucan and *Euglena* on RAW264.7 macrophages, as well as the effects of higher concentrations of these agents in a mouse model. Moreover, we focused on cytokine secretion by activated macrophage cells and in mouse models by measuring NO, iNOS, and cytokine production. In addition, to confirm the functions of β-glucan and *Euglena* in enhancing immunity, we employed the commonly used macrophage stimulation model and the cyclophosphamide (CCP)-induced mouse model. The mouse-derived RAW264.7 cell line is a macrophage cell line that exhibits inflammatory and immune reactions [16]. CCP, an alkylating agent widely used as a cancer therapeutic drug, causes side effects such as immunosuppression, damage to the spleen, and decreased numbers of lymphocytes [17]. This study therefore established a mouse model of immunosuppression by treating ICR mice with CCP to investigate the immune-enhancing effects of β-glucan (paramylon) and *Euglena*.

While many studies have demonstrated the anti-inflammatory effects of *Euglena*, or have focused on those of β-glucan, only a few have examined whether *Euglena* and β-glucan exert immune-enhancing effects. Therefore, we aimed to confirm that *Euglena* enhances immune responses in RAW264.7 macrophages in vitro and in a CCP-induced mouse model of immunosuppression in vivo by regulating immune-related cytokines.

## Materials and Methods

### Materials

Dulbecco’s phosphate-buffered saline (DPBS), fetal bovine serum (FBS), 1 M hydroxyethyl piperazine ethane sulfonic acid (HEPES) buffer, a penicillin‒streptomycin mixture, and high-glucose Dulbecco’s modified Eagle’s medium (DMEM) were obtained from Biowest (Nuaille’, France). Roswell Park Memorial Institute (RPMI) 1640 medium was obtained from Welegene (Korea). CCP, lipopolysaccharide (LPS), trypan blue, 2-mercaptoethanol, red blood cell lysis buffer, and concanavalin A (ConA) were purchased from Sigma‒Aldrich (USA).

### Preparation of β-Glucan and *Euglena*

β-Glucan and *Euglena* powder were provided by Daesang Corp., R&D Center (Korea). *Euglena gracilis* DSW1 cells (KCTC 13930BP) were incubated in a jar fermentor at 28°C for 6 days in medium supplemented with cyanocobalamin, glucose, DL-malic acid, dipotassium phosphate, and L-glutamic acid. After incubation, *Euglena* cells were collected and washed with water. The collected cells were then sterilized and dried. β-Glucan was prepared as follows: *Euglena* cells were cultured in the dark, harvested by centrifugation (4,000 ×*g*, 10 min) and washed twice with distilled water. The pH was adjusted to 12.5, and the samples were heated for 1 h at 60°C for extraction. The extract was collected by centrifugation (4,000 ×*g*, 10 min).

### Alkaline Treatment of β-Glucan and *Euglena*

Each β-glucan and *Euglena* powder sample was mixed with 0.5 mol/l NaOH to a weight per volume of 1% and stirred well to prepare a homogenous solution. Subsequently, 98% cold ethanol (twice the volume of the NaOH solution) was added, and the samples were centrifuged (12,000 ×*g*, 10 min, 4°C). The samples were resuspended in 40 ml of deionized water, and the addition of cold ethanol was repeated. After removal of the ethanol, 30 ml of deionized water was added, and the pellet was dissolved. The pH was adjusted to 7.0. During this procedure, 20%of the concentration was assumed lost [13].

### Cell Culture and Experimental Design

RAW264.7 cells were cultured in DMEM supplemented with 10% (v/v) FBS, 2% (v/v) 1 M HEPES, and 2% (v/v) penicillin‒streptomycin at 37°C in 5% CO_2_. The cells were seeded in 6-well plates (1×10^6^ cells/well), cultured for 4 h, and incubated with 100, 250, or 500 μg/ml *Euglena* (E100, E250, or E500) or 50, 125, or 250 μg/ml β-glucan (paramylon) (B50, B125, or B250) for 48 h.

### Nitric Oxide (NO) Synthase Assay

RAW264.7 cells were cultured for 4 h in black 96-well plates at a density of 1 × 10^5^ cells/well and then treated with β-glucan or *Euglena* for 48 h. NO production was measured using a Nitric Oxide Synthase Kit obtained from Sigma‒Aldrich (USA) according to the manufacturer’s protocol. The fluorescence intensity was measured using a SpectraMax i3x plate reader (Molecular Devices, USA) with an excitation wavelength of 490 nm and an emission wavelength of 520 nm.

### Animals and Study Designs

Five-week-old Institute of Cancer Research (ICR) male mice were acquired from Hana Biotech (Korea), and the protocol was approved by the Institutional Animal Care and Use Committee of Southeast Medi-Chem Institute (Receipt Number: SEMI-22-008). After a 6-day acclimation period, the mice were maintained under a 12-h dark-light cycle, pathogen free, and divided into groups (10 mice per group). The groups were as follows: normal control (Normal) group, 80 mg/kg body weight (B.W.) cyclophosphamide-only (CCP) group, CCP + 400 mg/kg B.W. β-glucan (B400) group, CCP + 400 mg/kg B.W. *Euglena* (E400) group, and CCP + 800 mg/kg B.W. *Euglena* (E800) group. The mice in the normal and CCP groups were orally administered saline, and those in the B400, E400 and E800 groups were orally administered 400 mg/kg B.W. β-glucan, 400 mg/kg B.W. *Euglena*, and 800 mg/kg B.W. *Euglena* for 19 days, respectively. On days 17-19, immunosuppression was established in all the groups except the normal group via the intraperitoneal injection of 80 mg/kg B.W. of CCP diluted in saline. The experimental design is shown in Fig. S1. Based on the first date of CCP administration (day 17), the changes in B.W. were calculated as follows:

Change in B.W. (%) = B.W. (g) on day 19/B.W. (g) on day 17 × 100

### Isolation of Splenocytes for Cell Culture

After the mice were sacrificed, the spleens were harvested, sterilized with saline, and weighed. The spleen index was calculated using the following formula:

Spleen index (mg/g) = Spleen weight (mg)/B.W. (g)

The spleens were filtered through a cell strainer (SPL Life Sciences, Korea). Red blood cells were removed, and the samples were washed with cold RPMI 1640 medium. The isolated splenocytes were seeded in 12-well plates at 1 × 10^6^ cells/well and incubated in RPMI 1640 medium supplemented with 5 μg/ml ConA or 3 μg/ml LPS as a mitogen for 24 h at 37°C in 5% CO_2_.

### RNA Extraction and Quantitative Reverse Transcription Polymerase Chain Reaction (RT‒qPCR)

Total RNA was extracted from splenocytes and RAW264.7 macrophages with TRIzol reagent (Life Technologies, USA) according to the manufacturer’s protocol. Total RNA was then synthesized into cDNA using a Transcriptor First-Strand cDNA Synthesis Kit (Roche, Switzerland). GAPDH was selected as the housekeeping gene for the normalization of relative mRNA expression. The primer sequences are presented in Table 1.

### Enzyme-Linked Immunosorbent Assay (ELISA)

Splenocyte culture supernatants were harvested, aliquoted, stored, and transferred to 96-well ELISA plates to measure the levels of secreted inflammatory cytokines. The concentrations of cytokines were measured using kits from R&D Systems (USA) according to the manufacturer’s protocol.

### Statistical Analysis

All data are presented as the means ± standard error of the mean (SEM). Statistical significance was analyzed by one-way ANOVA followed by Duncan’s multiple range test, and a *p*-value of less than 0.05 (*p* < 0.05) was considered to indicate statistical significance.

## Results

### β-Glucan and *Euglena* Enhanced iNOS Gene Expression and NO Production in Macrophages

**Fig. 1:**
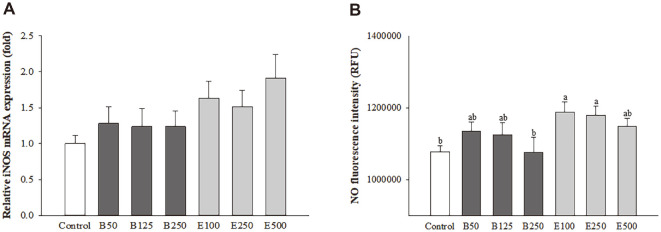
Effects of β-glucan and *Euglena* on iNOS (A) and NO (B) expression in macrophages. Data with different letters within a row are significantly different at *p* < 0.05, as determined by Duncan’s multiple range test. Values from large to small are arranged in alphabetical order.

iNOS levels and NO release in macrophages treated with 100, 250, or 500 μg/ml *Euglena* or 50, 125, or 250 μg/ml β-glucan were measured (Fig. 1). The mRNA expression of iNOS was increased by approximately 1.28-, 1.24-, and 1.24-fold in the B50, B125, and B250 groups, respectively, and 1.63-, 1.51-, and 1.91-fold in the E100, E250, and E500 groups, respectively, compared with that in the control group, but these differences were not significant (Fig. 1A). The levels of NO production are shown as relative fluorescence units (RFUs) (Fig. 1B). Treatment with β-glucan or *Euglena* increased the RFU values in most of the groups (B50 group, 105.25 ± 2.34%; B125 group, 104.39 ± 3.14%; E100 group, 110.20 ± 2.62%; E250 group, 109.38 ± 2.26%; and E500 group, 106.51 ± 1.99%) but not in the group treated with 250 μg/ml β-glucan (99.78 ± 3.85%) compared with that of the control group, and these values increased to greater extents in the groups treated with *Euglena*. The RFU value was significantly increased in the 100 μg/ml *Euglena* group (*p* = 0.0454). The results confirmed that the iNOS gene expression and NO production levels were increased in the *Euglena* groups compared with the control group.

### β-Glucan and *Euglena* Enhanced the TNF-α Gene Expression and Production Levels in Macrophages

**Fig. 2:**
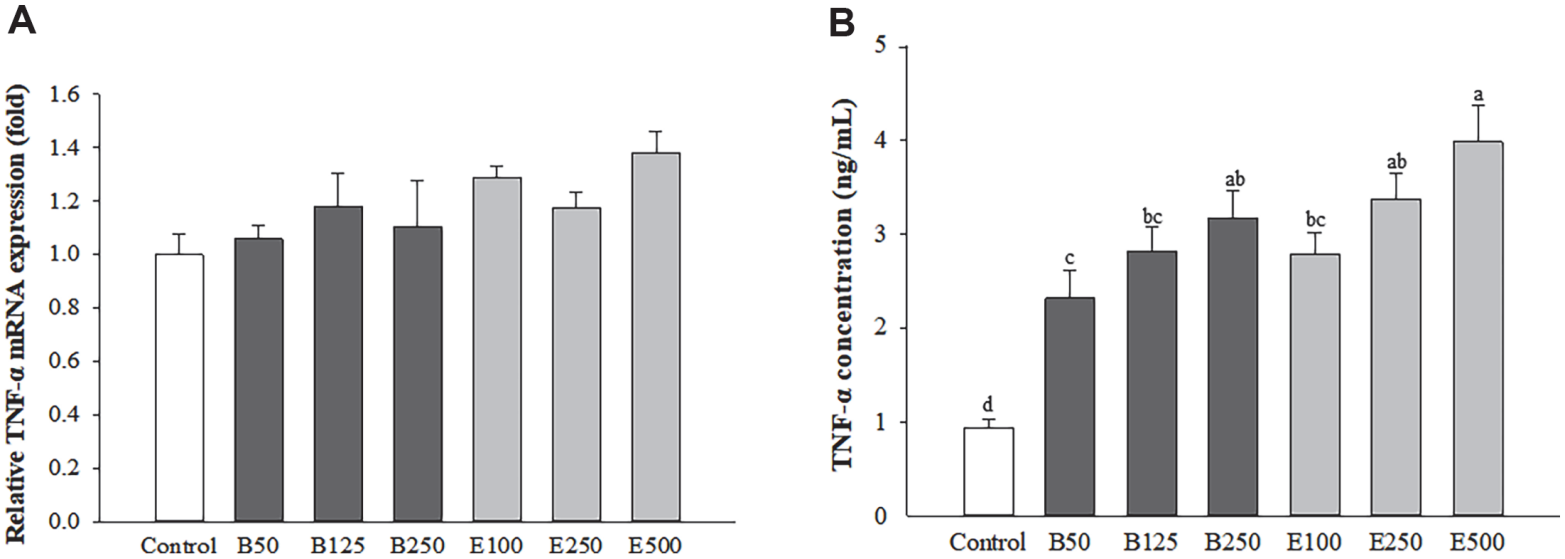
Effect of β-glucan and *Euglena* on TNF-α mRNA expression (A) and production (B) in macrophages. Data with different letters within a row are significantly different at *p* < 0.05, as determined by Duncan’s multiple range test. Values from large to small are arranged in alphabetical order.

The gene expression of TNF-α in macrophages leads to the release of this cytokine. Thus, we measured the TNF-α gene expression (Fig. 2A) and production levels (Fig. 2B) in RAW264.7 macrophages. The relative TNF-α mRNA expression was slightly increased by approximately 1.06-, 1.18-, and 1.10-fold in the B50, B125, and B250 groups, respectively, and 1.29-, 1.17-, and 1.38-fold in the E100, E250, and E500 groups, respectively, compared with that in the normal group. The TNF-α production level was increased in the β-glucan and *Euglena* groups compared with that in the normal group in a dose-dependent manner. The concentration of TNF-α was significantly increased in all the groups (B50 group, 2.49-fold; B125 group, 3.02-fold; B250 group, 3.41-fold; E100 group, 2.99-fold; E250 group, 3.62-fold; E500 group, 4.26-fold) compared with that in the normal group (*p* < 0.0001).

### Cyclophosphamide Decreased the Body Weight and Spleen Index of ICR Mice

As shown in Table 2, the B.W. of the mice in the four CCP-treated groups (CCP, 99.39 ± 0.34%; B400, 98.92 ± 0.51%; E400, 99.13 ± 0.52%; E800, 96.56 ± 1.04%) was lower than that of the mice in the normal group (103.29 ± 0.6%). The rate of B.W. loss (%) of the CCP group was substantially decreased (99.39 ± 0.34%) compared with that of the normal group (*p* < 0.0001). In addition, the spleen index of the CCP-treated groups was significantly lower (CCP, 2.00 ± 0.08; B400, 2.12 ± 0.08; E400, 2.10 ± 0.09; E800, 1.80 ± 0.05) than that of the normal group (3.42 ± 0.12) (*p* < 0.0001). As a result, CCP administration decreased the immune responses of ICR mice.

### β-Glucan and *Euglena* Upregulated the mRNA Expression of Factors That Enhance Immune Responses in Splenocytes

**Fig. 3:**
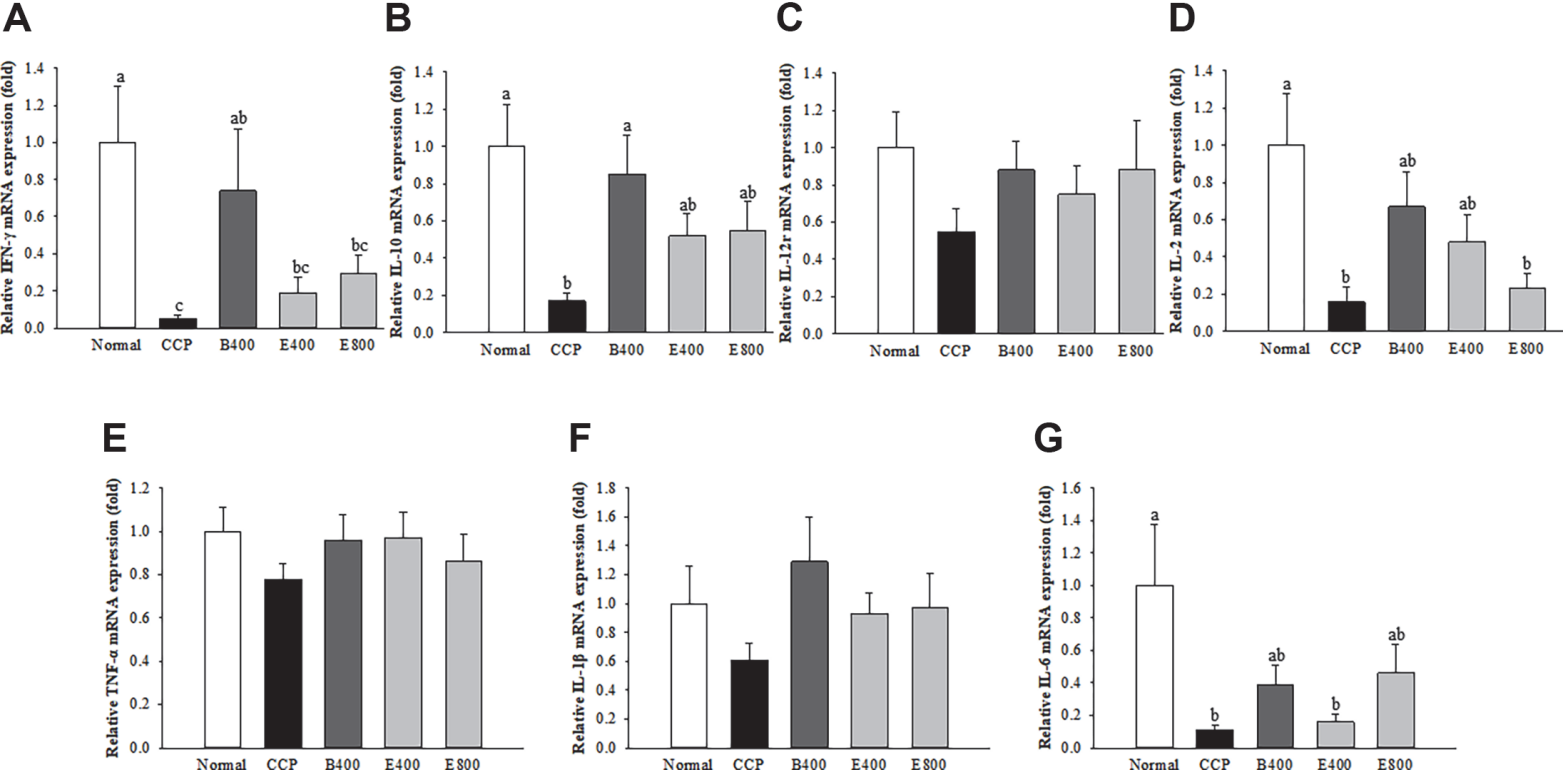
Effect of β-glucan and *Euglena* on the relative gene expression of IFN-γ (A), IL-10 (B), IL-12Rβ1 (C), IL-2 (D), TNF-α (E), IL-1β (F), and IL-6 (G), which are involved in the immune response, in ICR mice. Splenocytes were isolated and stimulated with 5 μg/ml concanavalin A (ConA) or 3 μg/ml lipopolysaccharide (LPS) as mitogens. Data with different letters within a row are significantly different at *p* < 0.05, as determined by Duncan’s multiple range test. Values from large to small are arranged in alphabetical order.

The IFN-γ, IL-10, IL-12Rβ1, and IL-2 expression levels in splenocytes from immunosuppressed ICR mice were upregulated by ConA, and the TNF-α, IL-1β, and IL-6 expression levels were upregulated by LPS. In particular, the levels of IFN-γ, IL-10, and IL-2 (Figs. 3A, 3B, and 3D) were significantly increased in the β-glucan-treated group compared with the CCP group. Compared with the CCP group, the β-glucan and *Euglena* groups exhibited restored (B400 group, 15.67-fold; E400 group, 3.99-fold; E800 group, 6.18-fold) IFN-γ mRNA expression (*p* = 0.0102). The mRNA expression of IL-10 was considerably different among the β-glucan, *Euglena* and CCP groups (B400 group, 5.08-fold; E400 group, 3.08-fold; E800 group, 3.27-fold) (*p* = 0.0095). The expression of IL-12Rβ1 (Fig. 3C) in both the β-glucan and *Euglena* groups was higher than that in the CCP group (B400 group, 1.62-fold; E400 group, 1.375-fold; E800 group, 1.61-fold), but the differences were not significant. In addition, the IL-2 mRNA expression levels in the B400 and E400 groups were significantly higher (B400 group, 4.16-fold; E400 group, 2.99-fold) than those in the CCP group (*p* = 0.016). The E800 group showed an approximately 1.43-fold increase in the mRNA expression of IL-2 compared with that in the CCP group, but the difference was not significant. Regarding LPS-stimulated cytokines, the expression levels of TNF-α and IL-1β (Figs. 3E and 3F) tended to be increased in the β-glucan and *Euglena* groups, but the differences were not significant. IL-6 mRNA expression (Fig. 3G) was significantly decreased in the CCP group compared with the normal group. However, the B400 and E800 groups showed increases of 3.69- and 4.34-fold, respectively, compared with the CCP group, but the difference between the E400 and CCP groups was not significant (*p* = 0.026).

## Discussion

β-Glucan has been considered the main component of *Euglena* [18]. Some studies have shown various health benefits of β-glucan, including its effects on modulating the immune response and reducing the incidence of infections [16, 19]. In this study, the immune-enhancing effects of *Euglena* and paramylon, which is a β-glucan component of *Euglena*, were examined in RAW264.7 macrophages and a CCP-induced ICR mouse model. By applying two different models, we compared the effects of *Euglena* on immunosuppression and macrophage activation.

Macrophages play an important role in mechanisms related to the immune system. RAW264.7 macrophages can produce iNOS, which mostly regulates the production of NO and cytokines such as TNF-α [19, 20]. iNOS prevents pathogen invasion by synthesizing NO in activated macrophages. NO exerts immune effects by increasing microbial damage [21, 22]. The cytokine TNF-α is known to protect against infectious pathogens. TNF is involved in systemic inflammation and is a protein whose plasma concentration depends on the degree of inflammation. TNF-α is produced by activated immune cells such as neutrophils, macrophages, neurons, CD4^+^ lymphocytes, NK cells, and mast cells. A major function of TNF is the regulation of inflammatory and immune responses by immune cells [23]. In previous studies, paramylon showed a similar pattern to that of increasing NO production, results which suggest that the production of NO and TNF-α by macrophages in the *Euglena* group leads to immune enhancement [19]. Therefore, this study confirmed that β-glucan and *Euglena* significantly enhanced immune responses by increasing the production of iNOS, NO, and TNF-α by macrophages.

CCP administration can disrupt the Th1/Th2 balance and decrease the number of immune cells such as monocytes and macrophages that regulate lymphocyte function. CCP leads to reduced proliferation and decreased numbers of T and B cells [24, 25]. As a result, decreases in the body weight, spleen and thymus indices, and damage to the intestinal mucosal barrier may occur in the body [26]. In the CCP-induced mouse model, immunosuppression was confirmed through reductions in the B.W. change and the spleen index. Moreover, both β-glucan and *Euglena* significantly increased the levels of various cytokines, such as IFN-γ, IL-10, and IL-2, induced by ConA in splenocytes derived from CCP-treated mice. However, the effect in splenocytes treated with LPS was relatively insignificant, with the exception of that on the cytokine IL-6. Thus, it seems likely that *Euglena* and β-glucan more effectively influenced the immune mechanisms related to T-cell activation by ConA than those related to B-cell activation by LPS. These results suggested that β-glucan and *Euglena* can effectively increase immunity via T-cell-mediated immune responses.

T and B lymphocytes play important roles in inducing effective immune responses [27]. ConA is a mitogen that stimulates only T lymphocyte proliferation. CD4^+^ and CD8^+^, which are clusters of differentiation, are surface markers of various subsets of T lymphocytes, namely, T helper (Th) and T cytotoxic lymphocytes, respectively. The CD4^+^ and CD8^+^ T lymphocyte proportions and the CD4^+^/CD8^+^ ratio are indicators of biological immune function [28, 29]. During T-cell activation, IFN-γ and IL-2 are essential cytokines produced by T cells to exert immunological effects [27, 30]. Th1 cells mostly enhance cell-mediated immunity, and Th2 cells enhance humoral immunity. The Th1/Th2 cytokine ratio is important in the immune system. Th1 cytokines include IFN-γ, IL-2, and IL-12. An increase in Th1 cytokine production is associated with the enhancement of T lymphocyte proliferation in adaptive immunity and supports cell differentiation in innate immunity. However, Th2 cytokines, which include IL-4 and IL-10, participate in the humoral immune response [31, 32]. LPS is a major stimulator of B lymphocytes and a representative B-cell mitogen. LPS induces not only the proliferation and differentiation of mature B cells, but also the secretion of IL-6 in B-cell lymphoma. Activated B cells have the ability to activate naïve CD4^+^ T cells. B cells can also contribute to the level of T-cell priming, and as antigen-presenting cells, are involved in antigen-specific Th2 immunity [33, 34]. IL-1β enhances the proliferation of follicular helper T (Tfh) cells, which help B lymphocytes differentiate into antibody-secreting cells and induce the production of IL-4 and IL-21, leading to a B-cell response [35, 36]. Additionally, Tfh cells increase B-cell-derived TNF-α production, which increases both macrophage recruitment and the expression of inflammatory cytokines such as TNF-α [37]. T and B cells cooperate to induce immune responses by the complex immune system.

A previous study demonstrated that *Euglena* enhances dectin-1 expression, which promotes innate immunity and induces inflammatory cytokine expression in lymphocytes [38]. These results show that *Euglena* might affect the immune response by T-cell-related cytokine expression in splenocytes and confirm its effect on the immune response in macrophage cells. Thus, in this study we sought to provide evidence supporting the immune-enhancing effect described in a previous study [38]. However, the detailed mechanisms underlying the immune response, including signaling pathways and T-cell mediation, have not been clearly elucidated. Therefore, further studies are needed to precisely elucidate the separated T-cell-mediated response and the overall mechanism through which *Euglena* powder enhances immunity.

In conclusion, *Euglena* powder might induce macrophage activation and T-cell-related cytokine expression via splenocytes, which consequently enhances the immune response. This finding can be helpful in developing functional ingredients to promote immune enhancement.

## Supplemental Materials

Supplementary data for this paper are available on-line only at http://jmb.or.kr.

## Figures and Tables

**Table 1:** Primer sequences used for qRT- PCR.

Gene	Forward (5’-3’)	Reverse (5'-3')
iNOS	ctttgccacggacgagac	tcattgtactctgagggctgac
IFN-γ	atctggaggaactggcaaaa	ttcaagacttcaaagagtctgagg
IL-10	cagagccacatgctcctaga	tgtccagctggtcctttgtt
IL-12Rβ1	ccccagcgctttagcttt	gccaatgtatccgagactgc
IL-2	gctgttgatggacctacagga	ttcaattctgtggcctgctt
TNF-α	tcttctcattcctgcttgtgg	ggtctgggccatagaactga
IL-1β	agttgacggaccccaaaag	agctggatgctctcatcagg
IL-6	gctaccaaactggatataatcagga	ccaggtagctatggtactccagaa
GHAPDH	aagagggatgctgcccttac	ccattttgtctacgggacga

iNOS, inducible nitric oxide synthase; IFN-γ, interferon-gamma; IL-10, interleukin-10; IL-12Rβ1, interleukin 12 receptor beta 1; IL-2, interleukin-2; TNF-α, tumor necrosis factor-alpha; IL-1β, interleukin 1 beta; IL-6, interleukin-6; and GAPDH, glyceraldehyde 3-phosphate dehydrogenase.

**Table 2:** Effect of β-glucan and *Euglena* on changes in the body weight and spleen index.

Group	Change in body weight (%)	Spleen index
Normal	103.29 ± 0.60^a^	3.42 ± 0.12^a^
CCP	99.39 ± 0.34^b^	2.00 ± 0.08^bc^
B400	98.92 ± 0.51^b^	2.12 ± 0.08^b^
E400	99.13 ± 0.52^b^	2.10 ± 0.09^b^
E800	96.56 ± 1.04^c^	1.80 ± 0.05^c^

Normal, normal control; CCP, treatment with CCP (80 mg/kg B.W.); B400, treatment with 400 mg/kg β-glucan and CCP (80 mg/ kg B.W.); E400, treatment with 400 mg/kg *Euglena* and CCP (80 mg/kg B.W.); E800, treatment with 800 mg/kg *Euglena* and CCP (80 mg/kg B.W.). The rate of body weight change after CCP injection is the ratio of the B.W. on day 19 compared with that on day 17. The data are presented as the means ± SEM. Significant differences (*p* < 0.05) between groups as determined by Duncan’s multiple range test are indicated with different letters.
