# Supplementary material for: Evaluation of the Effects of Euglena gracilis on Enhancing Immune Responses in RAW264.7 Cells and a Cyclophosphamide-Induced Mouse Model
Source: J Microbiol Biotechnol. 2023 Jan 20;33(4):493–9. doi: 10.4014/jmb.2212.12041 (PMC10164725; doi:10.4014/jmb.2212.12041)
Supplement: Supplementary file 1 [file jmb-33-4-493-supple.pdf]

J. Microbiol. Biotechnol.
https://doi.org/10.4014/jmb.2212.12041

Figure S1

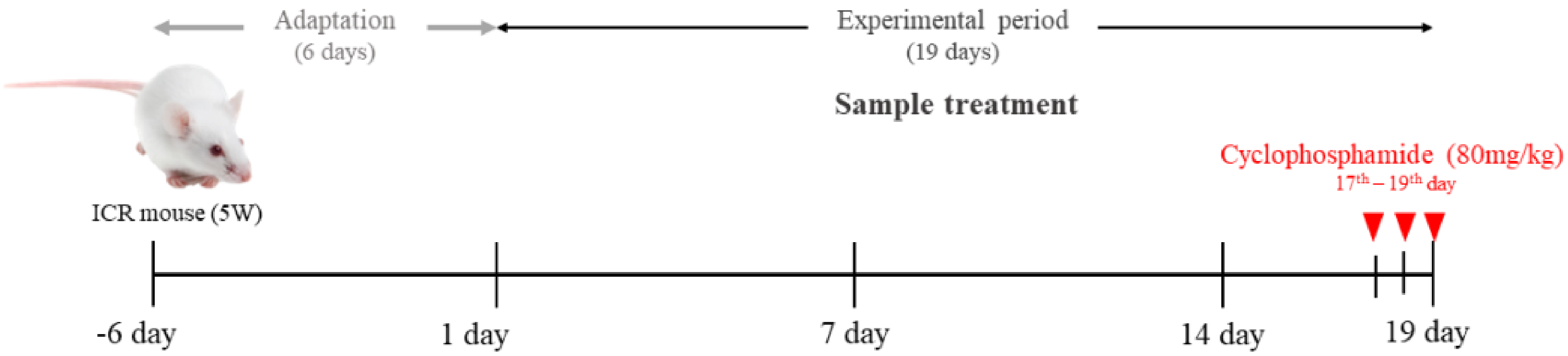

Fig. S1. Animal experimental design. After an adaptation period of six days, β-glucan or *Euglena* was orally administered daily to the mice in the B400, E400, and E800 groups for 19 days. CCP was intraperitoneally administered to the mice in all the groups, with the exception of those in the normal group, from days 17 to 19 before the animals were sacrificed.
